# Antifungal and Antiaflatoxigenic Methylenedioxy-Containing Compounds and Piperine-Like Synthetic Compounds

**DOI:** 10.3390/toxins8080240

**Published:** 2016-08-16

**Authors:** Young-Sun Moon, Won-Sik Choi, Eun-Sil Park, In Kyung Bae, Sung-Deuk Choi, Ockjin Paek, Sheen-Hee Kim, Hyang Sook Chun, Sung-Eun Lee

**Affiliations:** 1 School of Applied Biosciences, Kyungpook National University, Daegu 41566, Korea; space92@knu.ac.kr (Y.-S.M.); inj3773@naver.com (E.-S.P.); bae0601@korea.kr (I.K.B.); 2 Department of Life Science and Biotechnology, Soonchunhyang University, Asan 31538, Korea; wschoi@sch.ac.kr; 3 School of Urban and Environmental Engineering, Ulsan National Institute of Science and Technology, UNIST-gil 50, Ulsan 44919, Korea; sdchoi@unist.ac.kr; 4 Food Contaminants Division, National Institute of Food & Drug Safety Evaluation, Osong 28159, Korea; ojpaek92@korea.kr (O.P.); cinee@korea.kr (S.-H.K.); 5 Advanced Food Safety Research Group, BK21 Plus, School of Food Science and Technology, Chung-Ang University, Anseong 17546, Korea

**Keywords:** aflatoxin, *Aspergillus flavus*, methylenedioxy compounds, piperine, reverse transcription-PCR

## Abstract

Twelve methylenedioxy-containing compounds including piperine and 10 piperine-like synthetic compounds were assessed to determine their antifungal and antiaflatoxigenic activities against *Aspergillus flavus* ATCC 22546 in terms of their structure–activity relationships. Piperonal and 1,3-benzodioxole had inhibitory effects against *A. flavus* mycelial growth and aflatoxin B_1_ production up to a concentration of 1000 μg/mL. Ten piperine-like synthetic compounds were synthesized that differed in terms of the carbon length in the hydrocarbon backbone and the presence of the methylenedioxy moiety. In particular, 1-(2-methylpiperidin-1-yl)-3-phenylprop-2-en-1-one had potent antifungal and antiaflatoxigenic effects against *A. flavus* up to a concentration of 1 μg/mL. This synthetic compound was remarkable because the positive control thiabendazole had no inhibitory effect at this concentration. Reverse transcription-PCR analysis showed that five genes involved in aflatoxin biosynthesis pathways were down-regulated in *A. flavus*, i.e., *aflD*, *aflK*, *aflQ*, *aflR*, and *aflS*; therefore, the synthetic compound inhibited aflatoxin production by down-regulating these genes.

## 1. Introduction

Fungal infections are widespread in cereal crops, and severe contamination by fungal toxins, including aflatoxins, causes trade issues between countries [1,2,3,4]. The regulations for aflatoxin in cereals vary between countries, and the new maximum aflatoxin levels in the EU for corn and rice are <2 μg/kg aflatoxin B_1_ and a total aflatoxin content of 4 μg/kg, except for unprocessed maize and rice, which are 5 and 10 μg/kg, respectively [5]. Other foodstuffs, including almonds, pistachios, and apricot kernels, are limited to 8 μg/kg of aflatoxin B_1_ and a total aflatoxin content of 10 μg/kg [5]. Therefore, methods for reducing aflatoxin contamination have been developed in many countries, especially those that conduct regular monitoring of aflatoxin contamination in cereals [6,7,8].

Detoxification and decomposition of aflatoxins with organic acids reduces the residual aflatoxin contents of foodstuffs, where this method includes heating the crop [8,9]. Detoxification methods using enzymes, such as laccase and manganese peroxidase, have also been suggested for reducing aflatoxin contamination [10,11]. Physical and chemical methods, such as ozone and gamma-irradiation, can also remove aflatoxins from contaminated foodstuffs in an efficient manner [12,13]. The microbiological detoxification of aflatoxins has been studied comprehensively using *Rhodococcus* strains and yeasts [14,15]. Growth inhibition of aflatoxin-producing fungi, such as *Aspergillus flavus* and *A. parasiticus*, by microbes has been demonstrated successfully as a method for reducing aflatoxin contamination [16,17]. However, for some products, the detoxification processes may enhance their toxicities compared with those of the parent structures. Therefore, toxicity tests need to be determined after various treatments with structural elucidation of products [18].

Chemical control using currently available fungicides is one of the most efficient ways of preventing mycotoxigenic fungal growth and reducing mycotoxin contamination [19]. However, resistance to fungicides is well-documented throughout the world, and it threatens food security and human health [20,21].

The rise of fungal resistance necessitates the development of new methods for controlling mycotoxigenic fungi, and naturally occurring compounds, including essential oils, have been highlighted as alternative fungicides for reducing aflatoxin production [22,23,24]. Some isolated natural compounds have also been used to treat *A. flavus* growth and reduce aflatoxin production [25]. Piperlongumine, piperine, pipernonaline, and piperoctadecalidine exhibit fungicidal activities against *A. flavus* WRRC 3-90-42, and piperonal has a specific inhibitory effect against aflatoxin B_1_ biosynthesis [25,26]. Methylenedioxy moiety-containing compounds are abundant in *Piper* fruits (black pepper) and they are known to be inhibitors of cytochrome P450s [27,28]. Newly synthesized compounds derived from naturally occurring chemicals have also been suggested as compounds that could be used to control *Aspergillus* spp [29].

In this study, two methylenedioxy-containing compounds identified from *Piper nigrum*, piperonal and piperine, were investigated to determine their antiaflatoxigenic effects on aflatoxin production by *A. flavus*. Piperine was then used as a lead compound to synthesize various compounds containing the methylenedioxy moiety, and 10 piperine-like synthetic compounds were evaluated in terms of their structure–inhibitory activity relationships.

## 2. Results and Discussion

Thiabendazole is generally used in agriculture to control fungal infections in crop plants. In this study, we used thiabendazole as a positive control for comparison with the test compounds. We found that 1,3-benzodioxole exhibited antifungal activity at 1000 μg/mL, and kept some fungicidal activity at 100 μg/mL against *A. flavus* (Table 1). Similarly, methylenedioxy-containing compounds exhibited antifungal activities at 1000 μg/mL, but they lost most of their fungicidal effects at 100 μg/mL, except for methylenedioxy phenylacetic acid (Table 1). Thiabendazole achieved ca. 95% mycelial growth inhibition at 5 μg/mL. Piperine had very weak antifungal activities against *A. flavus* at 1000 μg/mL, and piperonal and sesamol obtained moderate antifungal effects against *A. flavus* at 1000 μg/mL. As shown in Table 1, we also determined the rate of aflatoxin production inhibition. Thiabendazole strongly inhibited the production of aflatoxins B_1_, B_2_, and G_2_ at 5 μg/mL, but aflatoxin G1 production was not inhibited at the same concentration. This indicates that thiabendazole inhibits the mycelial growth of *A. flavus* and the production of aflatoxins B_1_, B_2_, and G_2_ at 5 μg/mL, but not G_1_. 1,3-Benzodioxole had different inhibitory patterns where it controlled the production of four different aflatoxins at 100 μg/mL (Table 1). Piperine had a concentration-dependent inhibitory effect on aflatoxin production where it strongly inhibited aflatoxins B_1_, B_2_, and G_1_ at 3000 μg/mL, whereas it inhibited aflatoxin G_2_ at 1000 μg/mL. This difference may be attributable to the various inhibitory effects of piperine on aflatoxin production in *A. flavus*. By contrast, sesamol enhanced aflatoxin B_2_ production. According to these results, sesamol could inhibit mycelial growth, but the living mycelium produced more aflatoxins compared with the control group.

We found that methylenedioxy-containing compounds, including piperonal and piperine, had moderate inhibitory effects on the growth of *A. flavus* mycelia and aflatoxin B_1_ production (Table 1). The structure of the methylenedioxy-containing compounds used in this study contained 1,3-benzodioxole and its antiaflatoxigenic activity was the strongest of the methylenedioxy-containing compounds that we tested. In sesamol, the hydrogen in the compound is replaced by a hydroxyl moiety on the 1,3-benzodioxole, which decreased the antiaflatoxigenic activity compared with 1,3-benzodioxole (Table 1). Other replacement reactions also decreased the antiaflatoxigenic activities.

Methylenedioxy functional group-containing compounds, such as piperonal and piperine, have been identified as compounds that could potentially control aflatoxin contamination in foodstuffs [25,27,30]. Piperine is a major alkaloid found in *Piper* plants [31,32] which has an inhibitory effect on aflatoxin B_1_ biosynthesis and the growth of *A. flavus* mycelia at a concentration of 0.7% (*w*/*v*) [25]. Recently, Park et al. [25] showed that piperonal, one of the major compounds in *P. nigrum* essential oil, inhibited aflatoxin B_1_ production and it diverted the aflatoxin B_1_ biosynthetic route to aflatoxin G2 production. These findings improve our understanding of the relationship between chemical inhibition and aflatoxin biosynthesis.

**Figure 1:**
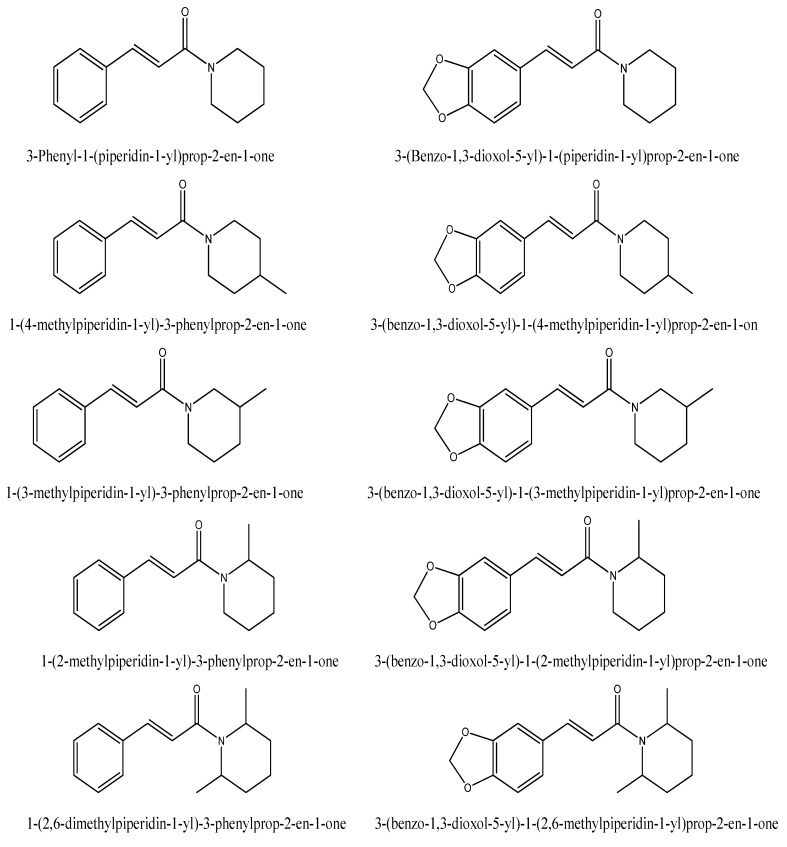
Piperine-like synthetic compounds used in this study.

Among the 10 piperine-like synthetic compounds (Figure 1), we found that 1-(2-methylpiperidin-1-yl)-3-phenylprop-2-en-1-one (**1**) and 3-(benzo-1,3-dioxol-5-yl)-1-(2-methylpiperidin-1-yl)prop-2-en-1-one exhibited antifungal activities against *A. flavus* at the concentration of 1000 μg/mL (Table 2), these antifungal activities decreased dramatically ten times less concentration than the initial concentration. Interestingly, 1-(2-methylpiperidin-1-yl)-3-phenylprop-2-en-1-one had potent antiaflatoxigenic activity up to 1 μg/mL (Table 2, Figure 1).

Piperine is a piperidine alkaloid that contains the methylenedioxy moiety in its structure. When the methylenedioxy moiety and dienes were removed from the structure of piperine, 1-(2-methylpiperidin-1-yl)-3-phenylprop-2-en-1-one was produced, which had moderate antifungal activities against *A. flavus*, but it had a potent antiaflatoxigenic effect against aflatoxin B_1_ when the concentration was as low as 1 μg/mL (Table 2).

**Figure 2:**
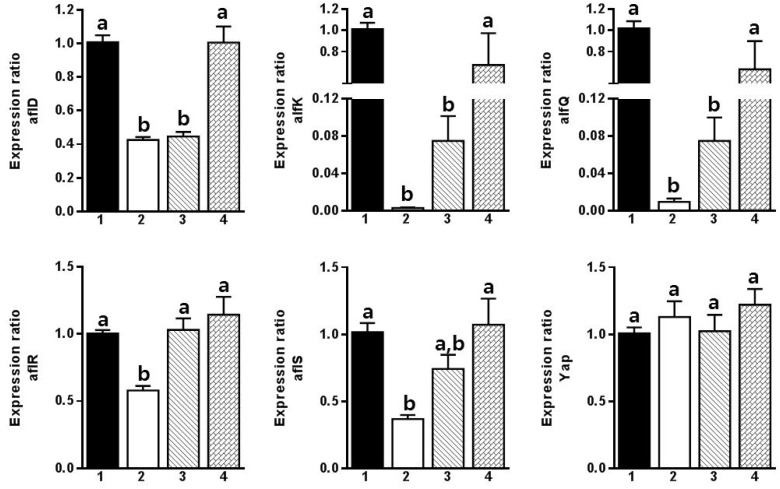
RT-PCR results obtained for six genes involved in aflatoxin biosynthesis (*aflD*, *aflK*, *aflQ*, *aflR*, *aflS*, and *yap*), which were regulated by 1-(2-methylpiperidin-1-yl)-3-phenylprop-2-en-1-one (**1**). 1, Control; 2, 1000 μg/mL of **1**; 3, 100 μg/mL of **1**; 4, 10 μg/mL of **1**.

RT-PCR analyses showed that 1-(2-methylpiperidin-1-yl)-3-phenylprop-2-en-1-one had dose-dependent inhibitory effects on the expression of *aflD*, *aflK*, *aflQ*, *aflR*, and *aflS* (Figure 2). It is likely that this compound directly blocks the aflatoxin biosynthesis pathway by inhibiting the aflatoxin biosynthesis transcription factors *aflR* and *aflS*.

The inhibitory mode of action was determined using RT-PCR (Figure 2). Two transcription factors, *aflR* and *aflS*, are known to control aflatoxin biosynthesis [33]. Our results showed that 1-(2-methylpiperidin-1-yl)-3-phenylprop-2-en-1-one inhibited the expression of the *aflR* and *aflS* genes, and three other genes were also downregulated (Figure 2). Therefore, this compound may be a potential biopesticide that could control *A. flavus* and aflatoxin production. The toxicological properties and other fungicidal effects of these compounds on mycotoxin-producing fungi need to be studied in future research.

## 3. Materials and Methods

### 3.1. Chemicals

Aflatoxins B_1_, B_2_, G_1_, and G_2_, and the chemicals 1,3-benzodioxole, eugenol, and methyleugenol were obtained from Sigma-Aldrich (St. Louis, MO, USA). Piperonal and piperine were isolated from *Piper nigrum* fruits, where they were analyzed and confirmed based on a series of spectrometric analyses including gas chromatography-mass spectrometry, and ^1^H- and ^13^C-nuclear magnetic resonance spectroscopy. Piperonal and piperine were also purchased from Sigma-Aldrich and compared with the corresponding compounds isolated in our laboratory. Asarone, methylenedioxy aniline, methylenedioxy phenylacetic acid, methylenedioxycinnamic acid, methylenedioxyphenyl propionic acid, and piperonyl alcohol were also purchased from Sigma-Aldrich. Ten piperine-like synthetic compounds were synthesized by Prof. Won-Sik Choi (Soonchunhyang University, Asan, Korea): 3-phenyl-1-(piperidin-1-yl)-2-en-1-one, 3-(benzo-1,3-dioxol-5-yl)-1-(piperidin-1-yl)prop-2-en-1-one, 1-(4-methylpiperidin-1-yl)-3-phenylprop-2-en-1-one, 3-(benzo-1,3-dioxol-5-yl)-1-(4-methylpiperidin-1-yl)prop-2-en-1-one, 1-(3-methylpiperidin-1-yl)-3-phenylprop-2-en-1-one, 3-(benzo-1,3-dioxol-5-yl)-1-(3-methylpiperidin-1-yl)prop-2-en-1-one, 1-(2-methylpiperidin-1-yl)-3-phenylprop-2-en-1-one (**1**), 3-(benzo-1,3-dioxol-5-yl)-1-(2-methylpiperidin-1-yl)prop-2-en-1-one, 1-(2,6-dimethylpiperidin-1-yl)-3-phenylprop-2-en-1-one, and 3-(benzo-1,3-dioxol-5-yl)-1-(2,6-methylpiperidin-1-yl)prop-2-en-1-one. All of the other chemicals used in this study were of the highest analytical grade and the concentrations tested for each chemical against *Aspergillus flavus* ATCC 22546 were presented in Table S1.

### 3.2. Aflatoxin Analysis by High-Performance Liquid Chromatography (HPLC)

*A.* *flavus* spores equivalent to 10^6^ were inoculated into 25 mL of potato dextrose broth (Difco, Sparks, MD, USA) liquid culture medium, before adding one of the test compounds. After the addition of fungal spores and the test compound, the culture was incubated with shaking for 5 days at 25 °C.

At least three replicates were performed for each concentration. After incubation for 5 days, the complete medium was used to determine the growth rates by measuring the mycelial and sclerotial dry weights using filter papers, and analyses of aflatoxin B and G type mycotoxin were performed using HPLC [25]. The arithmetic means were calculated based on three replicates. Dimethyl sulfoxide (DMSO) and thiabendazole were used as negative and positive control, respectively, for all experiments.

### 3.3. Total RNA Isolation and Quantitative Reverse Transcription-PCR (RT-qPCR)

*A.* *flavus* mycelia in liquid cultures were harvested carefully by filtering through a cell strainer (SPL Life Sciences Co. Ltd, Gyeonggi-do, Korea). The harvested mycelia were placed in a mortar and ground to a fine powder with an appropriate amount of liquid nitrogen. Total RNA was extracted from the *A. flavus* mycelia using QIAzol Lysis reagent (Qiagen Inc., Dusseldorf, Germany). The RNA extracts were quantified by determining the absorbance at both 260 and 280 nm using a μDrop^TM^ Plate system (Thermo Fisher Scientific Inc., Waltham, MA, USA) and the RNA was then evaluated qualitatively by agarose gel electrophoresis (1%) with ethidium bromide.

Complementary DNA (cDNA) was prepared using a Maxima First Strand cDNA Synthesis Kit (Thermo Fisher Scientific Inc., Waltham, MA, USA). The RNA extracts (2 μg) were used for compound synthesis. A Rotor-Gene SYBR Green PCR Kit (Qiagen Inc.) was used with 100 ng of cDNA for RT-qPCR analysis. Specific primers synthesized by Genotech (Daejeon, Korea) were used in this study to understand the relationship between aflatoxin biosynthesis and the chemicals tested, i.e., primers for *yap*, *aflR*, *aflS*, *aflK*, *aflD*, and *aflQ* 18S rRNA (Table 3). The amplification processes were performed as follows: denaturation at 95 °C for 30 s, annealing at 60 °C for 20 s, and elongation at 72 °C for 30 s. Forty rounds amplification were conducted according to the thermal cycling procedure with a postcycling step at 95 °C for 5 min. RT-qPCR was performed three times for each sample. Differences in gene expression after the addition of the test chemicals were calculated using the ΔC*t* method. The data were normalized against the 18S rRNA levels and the gene expression levels were compared.

### 3.4. Statistical Analysis

Experiments were performed three times and the data were expressed as the mean ± standard deviation. Statistically significant differences between experimental groups were determined by two-way ANOVA analysis with Tukey’s post-hoc test. Significant differences between experimental groups were accepted at *p* < 0.05. The statistical analyses were performed using Prism 6 software (GraphPad, San Diego, CA, USA).

## Figures and Tables

**Table 1:** Mycelial growth and aflatoxin (AF) production of *Aspergillus flavus* after treatment with various methylenedioxy-containing compounds.

Compound	Concentration (μg/mL)	Mycelial Growth Compared with the Control (%)	Aflatoxin Production Compared with the Control (%)			
			AFB1	AFB2	AFG1	AFG2
Thiabendazole (Positive control)	10	1.3 ± 2.3	- *	-	-	-
	5	6.90 ± 11.1	ND **	ND	35.2 ± 2.60	ND
	1	105 ± 26.1	>150	>150	131 ± 65.7	ND
1,3-Benzodioxole	1000	17.0 ± 3.10	0.03 ± 0.05	0.2 ± 0.4	2.0 ± 1.7	0.8 ± 1.3
	100	84.6 ± 5.90	25.2 ± 29.8	26.6 ± 17.9	0.4 ± 0.3	23.5 ± 13.9
Methylenedioxy phenylacetic acid	100	46.0 ± 19.7	-	-	-	-
	10	78.8 ± 18.4	-	-	-	-
Piperine	3000	133 ± 6.02	0.7 ± 0.1	1.6 ± 0.2	0.3 ± 0.6	55.2 ± 16.7
	1000	119 ± 6.70	39.1 ± 3.10	107 ± 27.1	21.6 ± 5.54	2.4 ± 0.053
Sesamol	1000	34.9 ± 15.1	140 ± 36.0	>150	38.4 ± 1.83	40.1 ± 34.7
	100	114 ± 8.92	-	-	-	-
Piperonal	1000	34.8 ± 1.17	10.5 ± 1.14	100 ± 46.2	21.3 ± 1.22	>150
	100 *	93.9 ± 5.06	45.0 ± 47.1	10.5 ± 6.12	0.30 ± 0.31	23.9 ± 19.8

* Not tested; ** ND, Not Detectable.

**Table 2:** Mycelial growth and aflatoxin (AF) production of *Aspergillus flavus* after treatment with piperine-like synthetic compounds.

Compound	Concentration (μg/mL)	Mycelial Growth Compared with the Control (%)	Aflatoxin Production Compared with the Control (%)			
			AFB1	AFB2	AFG1	AFG2
Thiabendazole (Positive control)	10	1.3 ± 2.3	- *	-	-	-
	5	6.90 ± 11.1	ND **	ND	35.2 ± 2.64	ND
	1	105 ± 26.1	>150.0	>150.0	131 ± 65.7	ND
1-(2-Methylpiperidin-1-yl)-3-phenylprop-2-en-1-one	1000	10.3 ± 17.8	ND	ND	35.51	ND
	100	64.3 ± 10.1	ND	ND	89.18	ND
	10	-	47.0 ± 2.45	69.4 ± 5.83	47.1 ± 6.08	ND
	1	-	38.0 ± 44.3	76.3 ± 55.9	122 ± 72.2	104 ± 58.0
3-(Benzo-1,3-dioxol-5-yl)-1-(2-methylpiperidin-1-yl)prop-2-en-1-one	1000	27.5 ± 5.43	ND	ND	64.7	55.9
	100	84.9 ± 31.8	96.4 ± 75.3	76.2 ± 55.9	122 ± 82.2	104 ± 57.9
	10	87.6 ± 18.6	>150	>150.00	>150.00	>150.00

* Not tested; ** ND, Not detectable.

**Table 3:** Gene-specific primers used for RT-qPCR.

Gene		Sequence
*yap*	Forward	5’ TGCAACCTCTCTACAAGCCG 3’
	Reverse	5’ CCGAAGTCTCGAGAAAGAGCC 3’
*aflR*	Forward	5’ GCACCCTGTCTTCCCTAACA 3’
	Reverse	5’ ACGACCATGCTCAGCAAGTA 3’
*aflS*	Forward	5’ GGAATGGGATGGAGATG 3’
	Reverse	5’ GGAATATGGCTGTAGGAAG 3’
*aflK*	Forward	5’ GAACTGCTTCAGTTGCCGTG 3’
	Reverse	5’ ACGAGGGTTCGTTTCTGGAC 3’
*aflD*	Forward	5’ TCCAGGCACACATGATGGTC 3’
	Reverse	5’ TGTGGATAACGAAGTGCCCC 3’
*aflQ*	Forward	5’ TTAAGGCAGCGGAATACAAG 3’
	Reverse	5’ GACGCCCAAAGCCGAACACAAA 3’
*18S rRNA*	Forward	5’ ATGGCCGTTCTTAGTTGGTG 3’
	Reverse	5’ GTACAAAGGGCAGGGACGTA 3’

## Supplementary Materials

The following are available online at www.mdpi.com/2072-6651/8/8/240/s1, Table S1: All chemicals and their concentrations tested in this study.
