# Supplementary material for: Antifungal and Antiaflatoxigenic Methylenedioxy-Containing Compounds and Piperine-Like Synthetic Compounds
Source: Toxins (Basel). 2016 Aug 16;8(8):240. doi: 10.3390/toxins8080240 (PMC4999856; doi:10.3390/toxins8080240)
Supplement: Supplementary file 1 [file toxins-08-00240-s001.pdf]

Supplementary Materials: Antifungal and Antiaflatoxigenic Methylenedioxy-Containing Compounds and Piperine-Like Synthetic Compounds

Young-Sun Moon, Won-Sik Choi, Eun-Sil Park, In Kyung Bae, Sung-Deuk Choi, Ockjin Paek, Sheen-Hee Kim, Hyang Sook Chun and Sung-Eun Lee

Table S1. All chemicals and their concentrations tested in this study

Chemicals	Tested Concentrations (μg/mL)	Regard
thiabendazole	10, 5, 1	Positive control, synthetic fungicide
1,3-benzodioxole	1000, 100	Natural compound, Sigma-Aldrich
sesamol	1000, 100	Natural compound, Sigma-Aldrich
eugenol	1000, 100	Natural compound, Sigma-Aldrich
methyleugenol	1000, 100	Natural compound, Sigma-Aldrich
Piperonal	1000, 100	Natural compound, Sigma-Aldrich, Isolated in my lab.
piperine	3000, 1000	Natural compound, Sigma-Aldrich, Isolated in my lab.
Asarone	1000	Natural compound, Sigma-Aldrich
methylenedioxy aniline	1000	Natural compound, Sigma-Aldrich
methylenedioxy phenylacetic acid	1000, 100, 10	Natural compound, Sigma-Aldrich
methylenedioxycinnamic acid	1000, 100	Natural compound, Sigma-Aldrich
methylenedioxyphenyl propionic acid	1000, 100	Natural compound, Sigma-Aldrich
piperonyl alcohol	1000	Natural compound, Sigma-Aldrich
kakuol	1000	Natural compound, Isolated in my lab.
3-phenyl-1-(piperidin-1-yl)-2-en-1-one	1000	Synthetic compounds
3-(benzo-1,3-dioxol-5-yl)-1-(piperidin-1-yl)prop-2-en-1-one	1000	Synthetic compounds
1-(4-methylpiperidin-1-yl)-3-phenylprop-2-en-1-one	1000	Synthetic compounds
3-(benzo-1,3-dioxol-5-yl)-1-(4-methylpiperidin-1-yl)prop-2-en-1-one	1000	Synthetic compounds
1-(3-methylpiperidin-1-yl)-3-phenylprop-2-en-1-one	1000	Synthetic compounds
3-(benzo-1,3-dioxol-5-yl)-1-(3-methylpiperidin-1-yl)prop-2-en-1-one	1000	Synthetic compounds
1-(2-methylpiperidin-1-yl)-3-phenylprop-2-en-1-one	1000, 100, 10, 1	Synthetic compounds
3-(benzo-1,3-dioxol-5-yl)-1-(2-methylpiperidin-1-yl)prop-2-en-1-one	1000, 100, 10	Synthetic compounds
1-(2,6-dimethylpiperidin-1-yl)-3-phenylprop-2-en-1-one	1000	Synthetic compounds
3-(benzo-1,3-dioxol-5-yl)-1-(2,6-methylpiperidin-1-yl)prop-2-en-1-one	1000	Synthetic compounds
